# Rules for Restoring the Drowned

**Published:** 1856-10

**Authors:** Marshall Hall


### RULES FOR RESTORING THE DROWNED.

BY DR. MARSHALL HALL, M. D., F. R. S.

The following rules are the *result* of half a year's investigation of Apnœa and Asphyxia—a subject which I propose to prosecute still further, knowing that truth only comes from long continued labor and research. I wish especially to put to the test of careful experiment, the correctness of the dogma, that if the heart has once ceased to beat, its action can never be restored—a dogma calculated to paralyze our efforts in many cases in which hope may really not be totally extint:

1. Treat the patient instantly, on the spot, in the open air, except in severe weather, freely exposing the face, neck and chest to the breeze.

2. Send with all speed for medical aid, and for articles of clothing, blankets, &c.

3. Place the patient gently on the face, with one arm under the forehead, so that any fluids may flow from the throat and mouth; and, without loss of time,—

TO EXCITE RESPIRATION,—

4. Turn the patient on his side, and
(i.) Apply snuff or other irritant to the nostrils.
(ii.) Dash cold water on the face previously rubbed briskly until it is warm.

If there be no success, again lose no time; but,—

TO IMITATE RESPIRATION,—

5. Replace the patient on his face; (when the tongue will then fall forward and leave the entrance into the windpipe free;) then,—

6. Turn the body gently but completely on the side and a little beyond (when inspiration will occur,) and then on the face, making gentle pressure along the back, (when expiration will take place,) alternately; these measures must be repeated deliberately, efficiently, and perseveringly, fifteen times in the minute, only; meanwhile,—

TO INDUCE CIRCULATION AND WARMTH,—

continuing these measures,—

7. Rub the limbs upwards, with firm pressure and with energy, using handkerchiefs, &c., for towels.

8. Replace the patient's wet clothing by such other clothing as can be instantly procured, each bystander supplying a coat, waistcoat, &c.

These rules are founded on physiology; and, whilst they comprise all that can be immediately done for the patient, exclude all apparatus, galvanism, the warm bath, &c., as useless, not to say injurious, especially the last of these; and all loss of time in removal, &c., as fatal.—*London Lancet.*

